# Predictive Value of Combined CRP and INR for Intracranial Hypertension in Cerebral Venous Thrombosis

**DOI:** 10.1002/iid3.70265

**Published:** 2025-10-15

**Authors:** Jiahui Yan, Manli Lu, Zhichao Huang, Yingying Xu, Yongjun Cao, Jianqiang Ni, Xia Zhang

**Affiliations:** ^1^ Department of Neurology and Suzhou Clinical Research Center of Neurological Disease The Second Affiliated Hospital of Soochow University Suzhou China; ^2^ Department of Neurology The First Affiliated Hospital of Soochow University Suzhou China

**Keywords:** cerebral venous thrombosis, combined effect, C‐reaction protein, international normalized ratio, intracranial hypertension

## Abstract

**Background:**

Intracranial hypertension (IH) is a frequently observed clinical manifestation of cerebral venous thrombosis (CVT), which reflects the severity of the disease. The gold standard of intracranial pressure (ICP) is through invasive lumbar puncture.

**Objectives:**

We aimed to develop a noninvasive model combining biomarkers and clinical features to predict IH in CVT patients, facilitating early risk stratification.

**Methods:**

The patients with CVT were consecutively enrolled in the Second Affiliated Hospital of Soochow University and the First Affiliated Hospital of Soochow University between January 2011 and June 2024, which were divided into two groups: CVT‐IH group and CVT + IH group based on ICP levels. Additionally, participants were further categorized into four groups according to the cut‐off of C‐reactive protein (CRP) and international normalized ratio (INR) by the receiver operating characteristic (ROC) curves. Logistic regression models were employed to calculate odds ratios (ORs) and 95% confidence intervals (CIs) of IH across the four subgroups.

**Results:**

157 individuals were finally included, 61 of whom had IH. Participants with CRP > 5.5 g/L or INR < 0.99 were more likely to experience IH. Those with high CRP and low INR conferred 9.778 times higher risk of IH compared with that of those with low CRP and high INR. Simultaneously adding CRP and INR to the basic model with established risk factors significantly improved risk discrimination and reclassification for IH of CVT patients.

**Conclusions:**

Combination of CRP and INR better predicted the occurrence of IH for CVT patients, which might provide a noninvasive way of assessing ICP of CVT patients.

## 1 Introduction

Cerebral venous thrombosis (CVT) is a relatively uncommon thrombotic disorder with a global incidence rate around 11.6 per 100,000 per year, which is higher in Asia [1, 2]. Despite advancements in the treatment of CVT over the years, it still carries a significant mortality rate ranging from 5% to 10%, posing substantial challenges for patients [3, 4, 5, 6, 7]. Therefore, the prompt identification and treatment of CVT are crucial [8]. Clinically, CVT presents with four main patterns: intracranial hypertension (IH), focal syndrome, diffuse encephalopathy, and cavernous sinus syndrome [9, 10]. Among these, IH has been reported in more than 60% of CVT patients. Previous research has highlighted that CVT patients with IH are more likely to experience persistent headaches, visual impairments, and other symptoms, indicating a correlation between IH and poor outcomes and prognosis [11, 12, 13, 14].

Lumbar puncture to measure the open pressure of cerebrospinal fluid (CSF) remains the most specific method for detecting IH. However, performing lumbar puncture in every patient at the early stage can be challenging due to factors such as disease severity and anticoagulation therapy, which is also invasive. Additionally, some studies have applied imaging features to estimate ICP, and even developed scoring systems. However, these methods often require precise scanning by radiologists, which may not always be readily available [15, 16]. Given these challenges, it is indeed necessary to establish more convenient methods for predicting IH in CVT patients. This would allow for quicker and more accessible assessments, facilitating early identification and timely intervention for better functional outcomes.

Previous studies have highlighted the significance of inflammation as a risk factor for the onset and development of CVT [17, 18, 19]. Additionally, coagulation indicators have been recognized as a vital role in the early identification of CVT [9]. Inflammatory and coagulation biomarkers, such as C‐reactive protein (CRP) and d‐Dimer, have been associated with the occurrence of acute/subacute CVT [20, 21], linked to poor outcomes [22, 23]. Moreover, it is important to note that IH in CVT may be partially attributed to the severe obstruction and narrowing of veins which might be associated with inflammation and coagulation disorders in CVT [20, 24]. In this context, these biomarkers can serve as potential valuable indicators for assessing the occurrence of IH in CVT patients.

However, there have been limited research specifically examining the predictive roles of these biomarkers in IH of CVT. And existing evidence suggests a mutual correlation between inflammation and coagulation systems [25]. Understanding this link between inflammation and coagulation factors provides a basis for exploring their potential combined predictive value in IH of CVT patients. Therefore, we suppose that combined inflammation and coagulation factors could better predict the occurrence of IH in CVT patients, which provides a noninvasive, operational, and convenient way to identifying IH high‐risk CVT patients in the early stage.

## 2 Methods

### 2.1 Study Design and Participants

**Figure 1:**
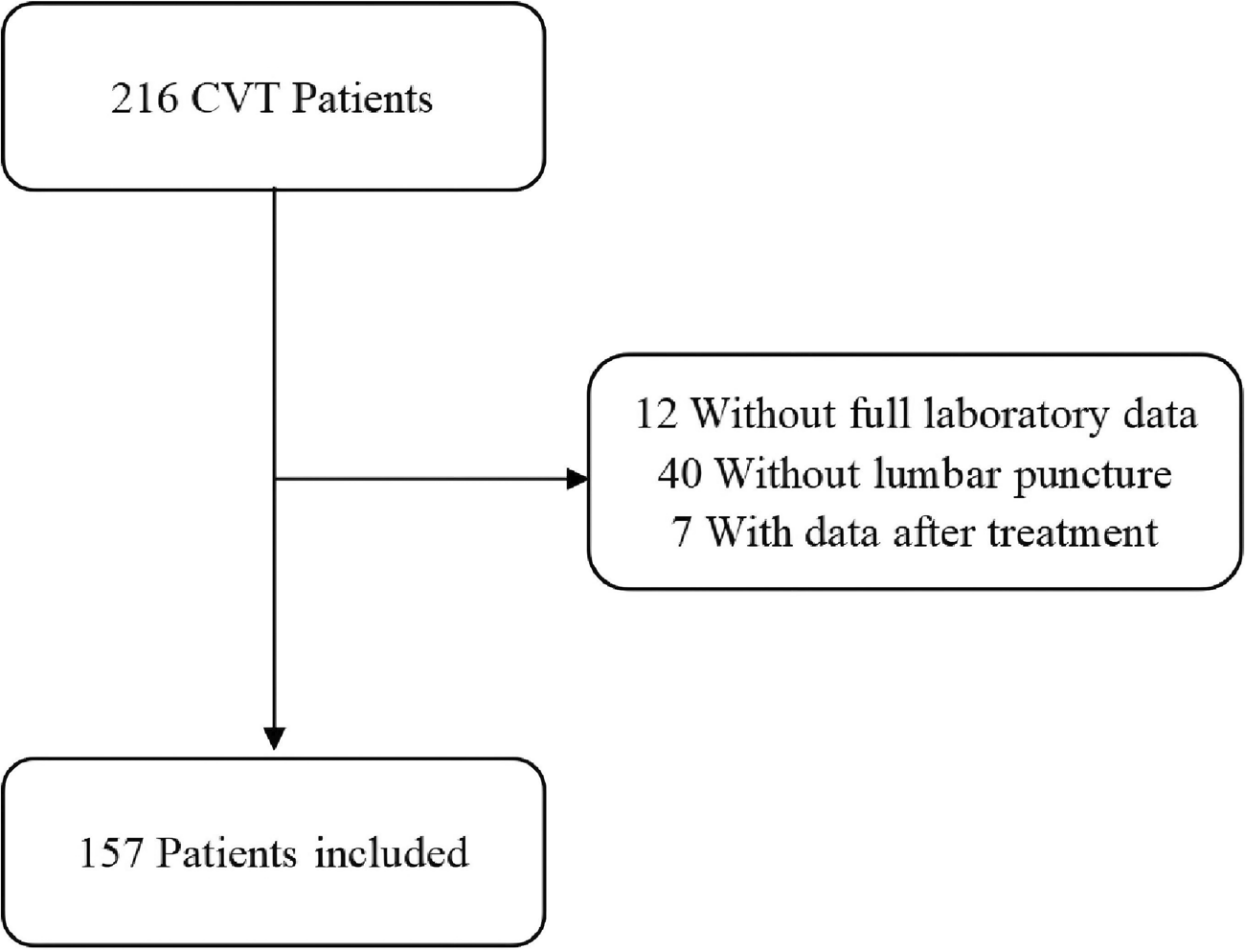
Patients' flow chart.

We performed a retrospective study of patients with a first episode of CVT from department of neurology, the Second Affiliated Hospital of Soochow University and the First Affiliated Hospital of Soochow University, the two main hospitals of Suzhou city, between January 2011 and June 2024. In the present study, the inclusion criteria were as follows: (1) age was > 14 years; (2) the diagnosis of CVT was confirmed by magnetic resonance imaging (MRI) combined with magnetic resonance venography (MRV), computed tomography venography (CTV) or digital subtraction angiography (DSA), following established diagnostic criterial [9]; (3) Lumbar puncture and all the laboratory tests were performed before any treatment; (4) valid information was available. Lumbar puncture was performed selectively in cases where: (1) clinical suspicion of intracranial hypertension (e.g., severe refractory headache) ‐ as measuring opening pressure directly informs therapeutic decisions for cerebrospinal fluid diversion or acetazolamide use; (2) alternative diagnoses needed exclusion (e.g., meningitis in febrile patients), particularly in early presentations before MRV, CTV or DSA confirmation. Patients without complete data of CRP, international normalized ratio (INR) or lumbar puncture were excluded. Also, laboratory tests or lumbar puncture performed after treatment or any infection was the exclusion criteria in this study (Figure 1). The study was approved by the Ethics Committee of the Second Affiliated Hospital of Soochow University (No. JD‐HG‐2024‐027). The data that support the findings of this study are available from the corresponding author upon reasonable request.

### 2.2 Data Collection, Laboratory Parameters and Clinical Assessment

All the participants underwent a standard collection of demographic characteristics, first symptoms, disease duration, location of thrombosis, risk factors of CVT including history of stroke and venous thrombosis disease, medication history (antiplatelet drugs, anticoagulation drugs, estrogen‐progesterone, puerperium, oral contraceptives,), sex‐specific factors (pregnancy), hereditary thrombophilia factors (protein C, S, or antithrombin III deficiency). ICP was estimated by measuring the open pressure of CSF during lumbar puncture before any treatment. An ICP of more than 250 mmH_2_O was defined as IH [26]. Besides, modified Ranking Scale (mRS) was performed evaluating the severity of disease at onset.

CRP was measured with the Mindray CRP‐M100 specific protein immune analyzer (Mindray CRP‐M100, Shenzhen, China) within 24 h of hospital admission before any treatment. Also, INR was analyzed with automatic coagulation analyzer (STA‐R Evolution, Stago, France). Other laboratory parameters included number of white blood cell (WCH), lymphocyte (L), monocyte (M), neutrophils (N), ratio of neutrophils and lymphocyte (NLR), d‐Dimer, and creatinine (CR). The estimated glomerular filtration rate (eGFR) was calculated by applying the Chronic Kidney Disease Epidemiology Collaboration (CKD‐EPI) equation with an adjusted coefficient of 1.1 for Chinese populations [27]:

141 *min (CR/к, 1)^a^ *max (CR/к, 1)^−1.209^*0.993^Age^*1.018[if female]*1.1, к is 0.7 for females and 0.9 for males, а is − 0.329 for females and − 0.411 for males, min indicates the minimum of CR/к or 1, and max indicates the maximum of CR/к or 1.

### 2.3 Statistical Analysis

The Shapiro‐Wilk test was used to test normality of continuous variables. The baseline characteristics of continuous variables were presented as means with SD or medians with interquartile ranges (IQR), and were compared using Student′s *t* test, Mann–Whitney *U* test or Kruskal–Wallis test. The categorical variables were expressed as a percentage and were compared using a Chi‐square test. All the patients were divided into two groups according to ICP: CVT with normal ICP (CVT‐IH) and CVT with IH (CVT + IH). We further examined the joint effects of CRP and INR. Each biomarker was dichotomized using the cut‐points identified by the receiver operating characteristic (ROC) curve. The participants were further divided into four groups: low CRP and high INR (LCHI), high CRP and high INR (HCHI), low CRP and low INR (LCLI), high CRP and low INR (HCLI). Logistic regression models were used to compute the odds ratios (ORs) and 95% confidence intervals (CIs) of IH across the four subgroups, separately. The multivariable‐adjusted model included demographic variables, symptoms, thrombotic locations, history, WBC, NLR, eGFR.

## 3 Results

### 3.1 Demographic and Clinical Assessment of Patients

A total of 216 individuals were recruited in our study, 40 of whom were excluded for the lack of lumbar puncture, 12 for the scanty of laboratory test, 7 due to the data collected after treatment. Thus finally 157 individuals were included (78 men and 79 women) with a medium age (IQR) of 37 (27, 48) years old. The median baseline mRS score was 2 (2, 3). Among them, 121 (77.0%) individuals reported headache; 17 (10.8%) suffered from seizure; 20 (12.7%) suffered from other venous thrombosis in the past, 18 (11.4%) had other risk factors of CVT such as medication history, pregnancy; the ICP of 61 (38.9%) was increased and the medium ICP was 220 (140, 280) mmH_2_O. The CVT + IH group demonstrated higher proportions of male patients and those with visual impairment or coma, while other baseline characteristics showed no significant differences between groups (Table 1).

**Table 1:** Demographic variables and clinical assessments between CVT patients with and without IH.

	CVT−IH (*n* = 96)	CVT + IH (*n* = 61)	All patients (*n* = 157)	*P*
Female sex, no. (%)	55 (57.2%)	24 (39.3%)	79 (50.3%)	0.028‡
Age (y)	35 (26, 49.75)	38 (27.5, 45)	37 (27, 48)	0.397*
mRS	2 (2, 3)	2 (2, 3)	2 (2, 3)	0.015*
Disease duration (d)	8 (4, 20)	6 (4, 14)	7 (4, 17)	0.066*
Neurological signs and symptoms at onset, no. (%)				
Headache	69 (71.8%)	52 (85.2%)	121 (77.0%)	0.052‡
Seizure	9 (9.3%)	8 (13.1%)	17 (10.8%)	0.462‡
Hemiplegia	8 (8.3%)	5 (8.1%)	13 (8.2%)	0.976‡
Hemisensory loss	10 (10.4%)	6 (9.8%)	16 (10.1%)	0.907‡
Mental disorders	4 (4.1%)	3 (4.9%)	7 (4.4%)	0.824‡
Visual impairment	5 (5.2%)	9 (14.7%)	14 (8.9%)	0.041‡
Coma	3, 3.1%	8, 13.1%	11, 7.0%	0.017‡
Location of thrombosis, no. (%)				
Superior sagittal sinus	32, 33.3%	25, 40.9%	57, 36.3%	0.331‡
Transverse sinus	61, 63.5%	44, 72.1%	105, 66.8%	0.265‡
Sigmoid sinus	26, 27.0%	21, 33.4%	47, 29.9%	0.327‡
Straight sinus	13, 13.5%	5, 8.1%	18, 11.4%	0.306‡
Inferior sagittal sinus	10, 10.4%	4, 6.5%	14, 8.9%	0.907‡
Cortex venous	13, 13.5%	13, 21.3%	26, 16.5%	0.202‡
Deep venous system	5, 5.2%	7, 11.4%	12, 7.6%	0.150‡
Sum of thrombotic locations	1 (1, 2)	2 (1, 3)	1 (1, 3)	0.024*
History of risks, no. (%)				
Smoking history	15 (15.6%)	13 (21.3%)	28 (17.8%)	0.364‡
History of venous thrombosis	12 (12.5%)	8 (13.1%)	20 (12.7%)	0.910‡
History of medication and other risk factors	13 (13.5%)	5 (8.1%)	18 (11.4%)	0.306‡
White blood cell (10^9^/L)	7.55 (6.23, 9.48)	8.60 (6.14, 10.40)	8.00 (6.17, 9.77)	0.211*
Lymphocyte (10^9^/L)	1.86 (1.39, 2.48)	1.76 (1.17, 2.21)	1.80 (1.30, 2.36)	0.124*
Monocyte (10^9^/L)	0.47 (0.37, 0.62)	0.53 (0.40, 0.68)	0.49 (0.39, 0.66)	0.238*
Neutrophils (10^9^/L)	5.65 (3.61, 7.00)	5.70 (4.00, 8.08)	5.2 (3.77, 7.38)	0.116*
NLR	2.70 (1.61, 4.63)	3.11 (1.84, 6.23)	3.04 (1.70, 4.71)	0.083*
CRP (g/L)	5.25 (3.01, 7.68)	7.00 (4.59, 15.03)	5.70 (3.31, 10.33)	0.002*
eGFR (ml/min/1.73 m^2^)	137.06 (121.00, 155.89)	129.83 (117.20, 155.74)	135.93 (117.64, 155.79)	0.378*
INR	1.04 (0.99, 1.10)	0.99 (0.95, 1.05)	1.02 (0.97, 1.08)	0.008*
d‐dimer (μg/mL)	1.02 (0.37, 1.73)	1.27 (0.50, 2.61)	1.05 (0.42, 1.99)	0.107*
CSF pressure (mmH_2_O)	160.00 (116.25, 200.00)	300.00 (270.00, 380.00)	220.00 (140.00, 280.00)	< 0.001*

Abbreviations: CRP, C‐reaction protein; CSF, cerebrospinal fluid; eGFR, estimated glomerular filtration rate; INR, international normalized ratio; mRS, modified Ranking Scale; NLR, ratio of neutrophils and lymphocyte.

* *P* values estimated using the Mann–Whitney *U* test.

^‡^ *P* values estimated using the Chi‐square test.

As for location of thrombosis, thrombosis most frequently involved the transverse sinus (66.8%) and superior sagittal sinus (36.3%), with 45.9% of patients exhibiting multiple thrombotic sites. Thrombosis distribution patterns did not differ significantly between groups (Table 1).

### 3.2 Independently Predictive Role of CRP and INR in IH

**Figure 2:**
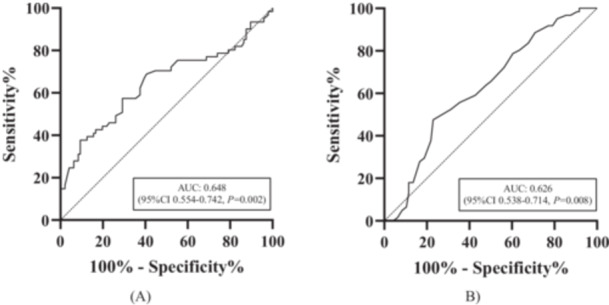
Cut‐off value of biomarkers: (A) CRP; (B) INR. CRP: C‐reaction protein: INR, international normalized ratio.

Compared with CVT‐IH group, CVT + IH group showed higher CRP (5.25 vs. 7.0, *p* = 0.002) and lower INR (1.04 vs. 0.99, *p* = 0.008) (Table 1). Based on the ROC curves, the AUC of CRP was 0.648 (95% CI 0.554–0.742, *p* = 0.002), the optimal cutoff value for CRP was identified as 5.5 g/L, exhibiting a sensitivity of 68.9%, a specificity of 59.4%, and a Youden′s index of 0.283. Similarly, the AUC of INR was calculated to be 0.626 (95% CI 0.538–0.714, *p* = 0.008). The best cutoff value for INR was found to be 0.99, offering a sensitivity of 47.5%, a specificity of 77.1%, and a Youden′s index of 0.246 (Figure 2). The ORs of CRP and INR without adjustment for IH were 1.114 (95% CI 1.050–1.182, *p* < 0.001) and 0.016 (95% CI 0.001–0.650, *p* = 0.029), respectively (Table 2). After adjusted for gender, age, mRS, duration, headache, seizure, visual disorder, coma, other symptoms, sum of thrombotic locations, smoking, history of thrombosis and others, WBC, NLR, eGFR, the ORs of CRP and INR for IH were 1.118 (95% CI 1.037–1.205, *p* = 0.004) and 0.002 (95% CI 0.001–0.228, *p* = 0.010) (Table 2).

**Table 2:** Odds ratios (OR) of IH according to CRP and INR separately.

Factors	Unadjusted OR (95% CI)	*P*	Adjusted OR1 (95% CI)	*P*	Adjusted OR2 (95% CI)	*P*
Gender (Female)	0.484 (0.251, 0.930)	0.029	0.806 (0.297, 2.192)	0.673	0.531 (0.196, 1.438)	0.213
Age	1.003 (0.983, 1.023)	0.786	0.977 (0.943, 1.012)	0.199	0.973 (0.938, 1.009)	0.140
mRS	1.532 (1.072, 2.188)	0.019	1.159 (0.643, 2.09)	0.624	1.171 (0.647, 2.118)	0.602
Disease duration	0.983 (0.964, 1.003)	0.103	0.988 (0.969, 1.006)	0.192	0.982 (0.959, 1.005)	0.130
Headache	2.261 (0.980, 5.215)	0.056	2.897 (0.911, 9.213)	0.072	2.988 (0.978, 9.123)	0.055
Seizure	1.459 (0.531, 4.013)	0.464	0.452 (0.084, 2.426)	0.354	0.707 (0.142, 3.524)	0.673
Visual impairment	3.150 (1.002, 9.899)	0.050	3.563 (0.901, 14.088)	0.070	6.653 (1.402, 31.578)	0.017
Coma	4.679 (1.190, 18.398)	0.027	2.690 (0.38, 19.044)	0.322	3.394 (0.527, 21.87)	0.199
Other symptoms	1.337 (0.519, 3.445)	0.547	2.052 (0.654, 6.441)	0.218	2.048 (0.654, 6.408)	0.218
Sum of thrombotic locations	1.235 (0.963, 1.584)	0.096	1.183 (0.862, 1.622)	0.299	1.199 (0.873, 1.646)	0.263
Smoking history	1.462 (0.642, 3.334)	0.366	2.220 (0.577, 8.537)	0.246	1.261 (0.330, 4.812)	0.734
History of venous thrombosis	1.057 (0.405, 2.755)	0.910	0.882 (0.251, 3.099)	0.845	0.964 (0.271, 3.428)	0.955
History of medication and other risk factors	0.570 (0.192, 1.688)	0.310	0.965 (0.269, 3.465)	0.956	0.894 (0.238, 3.355)	0.868
White blood cell	1.051 (0.950, 1.163)	0.336	0.880 (0.739, 1.048)	0.153	0.862 (0.719, 1.032)	0.106
NLR	1.063 (0.991, 1.141)	0.088	1.121 (0.996, 1.263)	0.059	1.172 (1.039, 1.322)	0.010
eGFR	0.996 (0.987, 1.006)	0.431	0.997 (0.984, 1.010)	0.643	0.994 (0.980, 1.007)	0.370
CRP	1.114 (1.050, 1.182)	< 0.001	1.118 (1.037, 1.205)	0.004	—	—
INR	0.016 (0.001, 0.650)	0.029	—	—	0.002 (0.001, 0.228)	0.010

*Note:* OR1 estimated from binary logistic regression models adjusted for gender, age, mRS, duration, headache, seizure, visual disorder, coma, other symptoms, sum of thrombotic locations, smoking, history of venous thrombosis and others, WBC, NLR, eGFR, CRP; OR2 estimated from binary logistic regression models adjusted for gender, age, mRS, duration, headache, seizure, visual disorder, coma, other symptoms, sum of thrombotic locations, smoking, history of venous thrombosis and others, WBC, NLR, eGFR, INR; mRS: modified Ranking Scale.

Abbreviations: CRP, C‐reaction protein; eGFR, estimated glomerular filtration rate; INR, international normalized ratio; NLR, ratio of neutrophils and lymphocyte; WBC, white blood cell.

### 3.3 Joint Effects of CRP and INR for Predicting IH

**Figure 3:**
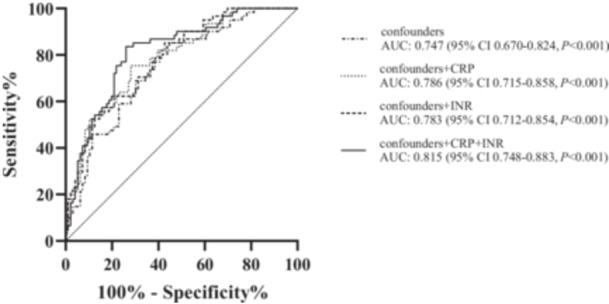
ROC curves of IH by confounders, CRP, and INR with confounders. Logistic confounding factors: gender, age, mRS, duration, headache, seizure, visual disorder, coma, other symptoms, sum of thrombotic locations, smoking, history of venous thrombosis and others, WBC, NLR, eGFR. mRS: modified Ranking Scale; NLR: ratio of neutrophils and lymphocyte; CRP: C‐reaction protein INR: international normalized ratio; eGFR: estimated glomerular filtration rate.

All the patients were divided into four groups according to the cutoffs of CRP and INR by ROC curves (Table 3). HCLI group had the worst mRS compared with other groups (*p* = 0.032) and shortest duration (*p* = 0.044). Compared with LCHI group, HCLI group were more likely to experience IH with the unadjusted OR of 9.778 (95% CI: 3.442‐27.780, *p* < 0.001) (Table 4). Moreover, the risk discrimination for IH by studied biomarkers was analyzed. The basic model included gender, age, mRS, duration, headache, seizure, visual disorder, coma, other symptoms, sum of thrombotic locations, smoking, history of thrombosis and other risk factors, WBC, NLR, eGFR. After adjusted for factors above, the OR of HCLI group was 11.857 (95% CI: 3.423–41.074, *p* < 0.001). The addition of CRP and INR to the basic model significantly improved discriminatory power, as AUC increased from 0.747 (95% CI: 0.670‐0.824, *p* < 0.001) to 0.815 (95% CI: 0.748–0.883, *p* < 0.001) (Figure 3).

**Table 3:** Demographic variables and clinical assessments according to the combination of CRP and INR.

	CRP− INR+ (*n* = 54)	CRP + INR+ (*n* = 52)	CRP− INR− (*n* = 22)	CRP + INR−(*n* = 29)	*P*
Female sex, no. (%)	32 (52.9%)	23 (44.2%)	10 (45.4%)	14 (48.2%)	0.431‡
Age (y)	40 (27, 50.5)	33 (26.25, 42.75)	36.5 (26.5, 46.75)	38 (27.5, 49.5)	0.559*
mRS	2 (1, 3)	2 (2, 3)	2 (2, 3)	2 (2, 3)	0.032*
Disease duration (d)	7.5 (4, 20)	6 (3, 10)	11 (5, 30)	10 (4, 21)	0.044*
Headache, no. (%)	38 (70.3%)	43 (82.6%)	17 (77.2%)	23 (79.3%)	0.497‡
Seizure, no. (%)	4 (7.4%)	7 (13.4%)	1 (4.5%)	5 (17.2%)	0.367‡
Visual impairment, no. (%)	3 (5.5%)	6 (11.5%)	1 (4.5%)	4 (13.7%)	0.465‡
Coma, no. (%)	2 (3.7%)	5 (9.6%)	0 (0.0%)	4 (13.7%)	0.161‡
Other symptoms, no. (%)	9 (16.6%)	2 (3.8%)	5 (22.7%)	4 (13.7%)	0.092‡
Sum of thrombotic locations	1 (1, 2)	1 (1, 2.75)	2 (1, 2.25)	2 (1, 3.5)	0.108*
Smoking history, no. (%)	8 (14.8%)	5 (9.6%)	7 (31.8%)	8 (27.5%)	0.056‡
History of thrombosis, no. (%)	7 (12.9%)	6 (11.5%)	1 (4.5%)	6 (20.0%)	0.384‡
History of other risk factors, no. (%)	5 (9.2%)	7 (13.4%)	4 (18.1%)	2 (6.8%)	0.565‡
White blood cell (10^9^/L)	7.85 (6.28, 9.34)	7.51 (6.13, 10.48)	7.7 (6.03, 9.87)	8.92 (7.26, 10.45)	0.343*
Lymphocyte (10^9^/L)	1.80 (1.37, 2.41)	1.89 (1.29, 2.54)	1.77 (1.29, 2.03)	1.76 (1.31, 2.43)	0.960*
Monocyte (10^9^/L)	0.44 (0.3, 0.54)	0.51 (0.4, 0.73)	0.52 (0.37, 0.67)	0.5 (0.39, 0.68)	0.017*
Neutrophils (10^9^/L)	5.10 (3.45, 7.03)	5.08 (3.71, 8.41)	4.86 (3.07, 7.4)	6.26 (4.15, 8.23)	0.437*
NLR	3.02 (1.71, 4.67)	2.65 (1.71, 4.75)	3.14 (1.59, 4.45)	3.18 (1.71, 6.23)	0.834*
CRP (g/L)	4.02 (1.39, 5.1)	10.33 (6.93, 15.08)	1.64 (0.7, 3.43)	9.66 (6.7, 15.03)	< 0.001*
eGFR (ml/min/1.73 m^2^)	130.29 (115.92, 154.66)	134.6 (115.14, 150.92)	147.98 (128.34, 174.85)	136.56 (116.13, 154.91)	0.231*
INR	1.05 (1.01, 1.1)	1.05 (1.02, 1.12)	0.93 (0.9, 0.97)	0.95 (0.92, 0.97)	< 0.001*
d‐dimer (μg/mL)	0.81 (0.28, 1.44)	1.22 (0.57, 2.3)	0.98 (0.38, 1.89)	1.6 (0.53, 2.16)	0.048*
CSF pressure (mmH_2_O)	160 (120, 220)	220 (136.25, 280)	232.5 (132.5, 292.5)	280 (225, 320)	< 0.001*

Abbreviations: CRP, C‐reaction proteine; CSF, cerebrospinal fluid; GFR, estimated glomerular filtration rate; INR, international normalized ratio; mRS, modified Ranking Scale; NLR, ratio of neutrophils and lymphocyte.

* *P* values estimated using the Kruskal–Wallis test.

^‡^ *P* values estimated using the Chi‐square test.

**Table 4:** Odds ratios (OR) of IH according to the combination of CRP and INR.

Factors	Unadjusted OR (95% CI)	*P*	Adjusted OR (95% CI)	*P*
Gender (Female)	0.484 (0.251, 0.930)	0.029	0.509 (0.180, 1.436)	0.202
Age	1.003 (0.983, 1.023)	0.786	0.984 (0.949, 1.021)	0.399
mRS	1.532 (1.072, 2.188)	0.019	1.314 (0.695, 2.483)	0.401
Disease duration	0.983 (0.964, 1.003)	0.103	0.976 (0.948, 1.005)	0.100
Headache	2.261 (0.980, 5.215)	0.056	2.856 (0.864, 9.438)	0.085
Seizure	1.459 (0.531, 4.013)	0.464	0.525 (0.096, 2.863)	0.457
Visual impairment	3.150 (1.002, 9.899)	0.050	3.148 (0.752, 13.174)	0.116
Coma	4.679 (1.190, 18.398)	0.027	3.285 (0.456, 23.687)	0.238
Other symptoms	1.337 (0.519, 3.445)	0.547	2.108 (0.638, 6.964)	0.221
Sum of thrombotic locations	1.235 (0.963, 1.584)	0.096	1.133 (0.810, 1.585)	0.464
Smoking history	1.462 (0.642, 3.334)	0.366	1.105 (0.263, 4.638)	0.891
History of thrombosis	1.057 (0.405, 2.755)	0.910	0.788 (0.208, 2.980)	0.725
History of other risk factors	0.570 (0.192, 1.688)	0.310	0.945 (0.253, 3.526)	0.933
White blood cell	1.051 (0.950, 1.163)	0.336	0.854 (0.710, 1.027)	0.094
NLR	1.063 (0.991, 1.141)	0.088	1.147 (1.016, 1.295)	0.026
eGFR	0.996 (0.987, 1.006)	0.431	0.509 (0.180, 1.436)	0.629
CRP + INR‐	9.778 (3.442, 27.780)	< 0.001	11.857 (3.423, 41.074)	< 0.001

Abbreviations: CRP,C‐reaction protein; CSF, cerebrospinal fluid; eGFR, estimated glomerular filtration rate; INR, international normalized ratio; mRS: modified Ranking Scale; NLR, ratio of neutrophils and lymphocyte.

*P* values estimated from binary logistic regression models adjusted for gender, age, mRS, duration, headache, seizure, visual disorder, coma, other symptoms, sum of thrombotic locations, smoking, history of venous thrombosis and others, WBC, NLR, eGFR.

## 4 Discussion

In the progress of CVT, IH is a common pathophysiological alteration that can lead to various symptoms and significantly impact patient outcomes and prognosis. Therefore, early detection of IH is of utmost importance. In our exploratory study, we enrolled 157 patients and observed a correlation between IH and higher levels of CRP and lower levels INR. After adjusting for confounding factors, our findings demonstrated that the risk of IH was 11 times higher in the HCLI group compared to that of LCHI group. Furthermore, our model exhibited ideal diagnostic value, with both good sensitivity and specificity. This indicates that the combination of CRP and INR can enhance the predictive value for IH in CVT patients. These findings highlight the potential utility of CRP and INR as convenient and available biomarkers for the early detection of IH in CVT, leading to improved management strategies and patient outcomes.

Previous studies focused on risk factors for IH in CVT have been more related to the location and volume of thrombosis. Harlof et al. found that patients with IH are often associated with higher thrombus volume and specific locations of thrombosis, such as superior sagittal sinus and transverse sinus thrombosis [16]. Ji et al. designed a novel score, based on MRV and CTV to predict IH, illustrating that IH was associated with higher thrombus burden [15, 28]. However, there are indeed few research on biomarkers for predicting IH in CVT. The present study demonstrated for the first time the prognostic effect of combined CRP and INR on IH in CVT patients, which was the strength of our study. By investigating the role of inflammation and coagulation factors, which can be readily obtained from emergency department assessments, we provide a more convenient and easier‐to‐perform approach for predicting IH in CVT patients. By identifying biomarkers that can be quickly and easily measured, our findings have potential practical implications in clinical guidelines and practice [9], enabling prompt intervention and improved patient outcomes.

Indeed, previous studies have reported associations between inflammation and CVT or other thrombosis, this process was also called ‘thrombo‐inflammation′ [29]. Inflammatory biomarkers, such as CRP, have been found to be associated with the onset and progression of CVT. The mechanism behind this association involves stimulation of platelet adhesion and responsiveness, promotion of the expression of P‐selectin on endothelial cell surfaces, increased tissue factor (TF) expression, decreased TF pathway inhibitor (TFPI) expression, and ultimate acceleration of thrombus growth by CRP [30]. Furthermore, Ji et al. reported that the acute and subacute phases of CVT exhibited higher neutrophil counts, lower NLR, and higher CRP levels compared to that of the chronic phase [20, 23]. Similar findings have been reported in Caucasian populations as well [21, 31]. Other inflammatory factors, such as platelet‐lymphocyte ratio (PLR), C‐reactive protein‐albumin ratio (CAR) and NLR, have also been found to be associated with CVT [19, 32]. In our study, we firstly pointed out higher levels of CRP was an independently risk factor for IH in CVT patients with odds ratio (OR) of 1.114 (unadjusted) and 1.118 (adjusted). These findings further support the role of inflammation in the pathogenesis of CVT and highlight the potential value of CRP as a biomarker for identifying IH in CVT patients.

Alternations of coagulation factors have been widely explored in CVT patients. d‐dimer has been recognized for its high sensitivity in identifying CVT patients, which is recommended to be tested in all suspected cases [9, 33]. Another relevant coagulation factor is prothrombin time (PT), which is assessed through clotting tests to monitor thrombotic conditions. However, INR is rarely investigated. INR is a standardized index derived from PT values obtained from different laboratories. PT reflects the activation of coagulation factors I, II, V, VII, and X. Factor V has been identified as influencing the formation of venous thrombosis [34, 35, 36]. Notably, factor V Leiden mutation has been confirmed as an independent risk factor for CVT [37, 38]. In our study, we firstly found that CVT patients with IH displayed lower INR values, with an unadjusted OR of 0.016 and an adjusted OR of 0.002 for IH. A lower INR indicates the activation of relevant coagulation factors, suggesting a higher risk of congestion. This finding aligns with the mechanisms involved in CVT pathogenesis and provides further insight into the relationship between coagulation factors and IH in CVT patients.

The exact pathogenetic mechanism of IH after CVT remains unknown. However, there are various mechanisms and complications that can contribute to elevated ICP secondary to CVT, which include venous stasis, parenchymal infarcts and edema, intracranial hemorrhage, and impaired CSF resorption [39, 40]. When a thrombus forms in the cerebral veins, it can expand and obstruct large draining venous sinuses. This, in turn, creates physiological back pressure in the venous system, leading to reduced drainage of CSF and subsequent intracranial hypertension. The underlying mechanism of our findings may be attributed to an overreaction of the inflammation and coagulation processes, which can synergistically contribute to congestion in small veins. Additionally, previous studies confirmed a bidirectional relationship between inflammation and coagulation, which suggests that inflammation may lead to activation of coagulation and vice versa [25]. Tissue factor and factor VII play a role in this reciprocal relationship [41]. While further research is needed to fully understand the precise mechanisms involved, our study suggests that the link among inflammation, coagulation, and venous congestion may play a significant role in the development of IH following CVT.

### 4.1 Study Limitations

Our study has successfully identified a practical and rapid approach to predict IH, allowing for the early implementation of dehydration as a potential treatment strategy and ultimately improving patient outcomes. However, it is important to acknowledge the limitations of our study. Firstly, our study was conducted retrospectively, which resulted in a relatively small sample size. Furthermore, there were some patients who had to be excluded from the study due to missing data in their laboratory test results. Secondly, although previous research has shown associations between inflammation factors and different phases of CVT, we were unable to divide the patients into specific phases due to the small population size of our study. Thirdly, the relatively high rate of lumbar puncture may introduce selection bias by predominantly including symptomatic IH patients while potentially overlooking milder presentations. Future studies could incorporate noninvasive IH markers (e.g., optic nerve sheath diameter ultrasound) to mitigate this limitation. Moreover, the number of inflammatory factors studied in our research was restricted, which may have impacted the comprehensiveness of our conclusions. Lastly, it is important to note that further follow‐up and exploration are essential to gain more insights into the long‐term effects and outcomes of IH in CVT patients.

Future research should focus on several key areas to further validate and extend our findings. First, multicenter studies with larger sample sizes are needed to enhance the statistical power and generalizability of the predictive model. Second, longitudinal investigations should consider the different phases of CVT (acute, subacute, and chronic), as their predictive values may vary during disease progression. Third, expanding the biomarker profile to include other inflammatory markers (such as IL‐6, TNF‐α, and erythrocyte sedimentation rate) could improve the model′s accuracy and provide deeper insights into the pathophysiology of IH in CVT. Finally, prospective intervention studies are warranted to evaluate whether early therapeutic strategies based on these biomarkers can effectively prevent IH and improve clinical outcomes. These comprehensive investigations will not only strengthen our understanding of IH mechanisms in CVT but also facilitate the development of personalized management strategies for better patient care.

## 5 Conclusion

CVT patients with IH exhibited higher levels of CRP and lower INR. Combined CRP and INR may have a stronger predictive value for IH in CVT patients. Further research is needed to validate the predictive value of this biomarker combination and to gain a deeper understanding of IH in the progress of CVT.

## Author Contributions

**Jiahui Yan:** writing – original draft, conceptualization, formal analysis, data curation, visualization. **Manli Lu:** writing – original draft, conceptualization, formal analysis, validation. **Zhichao Huang:** methodology, software, formal analysis. yingying xu: software, formal analysis, validation. **Yongjun Cao:** funding acquisition, supervision. **Jianqiang Ni:** funding acquisition, supervision, investigation. **Xia Zhang:** writing – review and editing, project administration, resources, investigation, funding acquisition.

## Conflicts of Interest

The authors declare no conflicts of interest

## Supporting information

STROBE checklist.

## Data Availability

The data that support the findings of this study are available from the corresponding author upon reasonable request.
